# Progress Toward Poliomyelitis Eradication — Afghanistan, January 2023–September 2024

**DOI:** 10.15585/mmwr.mm7349a4

**Published:** 2024-12-12

**Authors:** Colleen Mone Hardy, Mandeep Rathee, Sumangala Chaudhury, Mufti Zubair Wadood, Fazal Ather, Elizabeth Henderson, Maureen Martinez

**Affiliations:** ^1^Global Immunization Division, Global Health Center, CDC; ^2^Polio Eradication Department, World Health Organization, Kabul, Afghanistan; ^3^Polio Eradication Department, World Health Organization, Geneva, Switzerland; ^4^Polio Eradication Department, World Health Organization, Amman, Jordan; ^5^Division of Viral Diseases, National Center for Immunization and Respiratory Diseases, CDC.

**Summary**

**What is already known about this topic?**

Wild poliovirus (WPV) type 1 (WPV1) circulation continues in Afghanistan as well as in neighboring Pakistan, the two remaining countries with ongoing endemic WPV transmission.

**What is added by this report?**

During 2024 (through September 30), Afghanistan reported 23 WPV1 cases, the highest number in 4 years. Poliovirus vaccination campaign coverage improved markedly with house-to-house vaccination; however, local authorities have reinstated restrictions on house-to-house vaccine administration.

**What are the implications for public health practice?**

New challenges continue to affect progress toward polio eradication in Afghanistan. Both nationwide house-to-house vaccination campaigns and strengthened routine childhood immunization are needed to reach every vulnerable child with vaccines in Afghanistan and provide additional pathways toward stopping transmission.

## Abstract

Since the Global Polio Eradication Initiative began in 1988, wild poliovirus (WPV) types 2 and 3 have been eradicated, and annual polio case numbers have decreased by >99.9%. WPV type 1 (WPV1) transmission remains endemic in Afghanistan and Pakistan, two countries that share a 1,600-mile (2,600-km) border. This report describes immunization and surveillance activities and progress toward polio eradication in Afghanistan during January 2023–September 2024. As of November 1, Afghanistan reported 23 WPV1 cases in 2024, with onset during January–September 30, 2024. During the 3 previous years, 12 WPV1 cases were reported, including six during 2023. In August 2021, the Taliban took control nationwide and allowed increased geographic access for poliovirus vaccination campaigns. Multiple challenges have affected polio eradication activities in Afghanistan, including mandated repatriation of approximately 1 million Afghans by Pakistan beginning in late 2023, the ongoing humanitarian crisis that limits international agency effectiveness, polio program constraints imposed by authorities, and increased restrictions on female participation in vaccination activities. House-to-house vaccination coverage reached 90%–98% of children during June–July 2024. Beginning in 2021, authorities had progressively lifted restrictions on house-to-house campaigns, but abruptly reverted to national restrictions in September 2024. Both nationwide house-to-house activities and strengthening of the routine childhood immunization program would help ensure that every vulnerable child is vaccinated and provide a pathway to polio eradication in Afghanistan.

## Introduction

After the Global Polio Eradication Initiative (GPEI) was launched in 1988, wild poliovirus (WPV) type 2 and type 3 were eradicated, and the annual incidence of poliomyelitis decreased by >99.9%; however, WPV type 1 (WPV1) transmission remains endemic in Afghanistan and neighboring Pakistan (*1*,*2*). In 2016, the global synchronized switch from use of trivalent oral polio vaccine (tOPV) (containing Sabin strain types 1, 2, and 3) to use of 3 doses of bivalent OPV (bOPV) (containing Sabin types 1 and 3) and introduction of ≥1 dose of inactivated poliovirus vaccine (IPV) (containing types 1, 2, and 3 antigens) occurred in the routine childhood immunization schedule (*3*). During 2021, Afghanistan introduced a second dose of IPV into the routine immunization schedule (*3*).

Afghanistan is administratively divided into eight regions, 34 provinces, and 400 districts. Since the Taliban takeover in 2021, the humanitarian crisis has worsened, and several challenges continue: further economic stagnation, food insecurity, poor water and sanitation systems, limited access to health care, and increased social restrictions on women (*4*,*5*). This ongoing crisis was further compounded when approximately 1 million Afghans were forced to return from Pakistan during October 2023 to early 2024 (*6*). Competing needs (e.g., water and sanitation, nutrition, and food security) can result in lower community prioritization of vaccination. The polio program partners implemented several integrated strategies to help address some of these needs.

During 2022–July 2024, Afghanistan progressively moved toward the global standard of nationwide house-to-house (door-to-door) vaccination campaigns. The house-to-house modality is a more effective means of reaching every child when compared with temporary fixed vaccination sites (fixed post with multiple posts serving a narrow population area, or fixed sites at mosques, a single post serving a wide population area) that require caregivers to bring their children to these locations.

## Methods

### Data Sources

Data were provided by the Afghanistan National Emergency Operation Center, the World Health Organization (WHO), and UNICEF, including data obtained from acute flaccid paralysis (AFP) surveillance, environmental surveillance (ES), and supplementary immunization activity (SIA)* effectiveness measures. GPEI monitors the sensitivity of case detection and investigation through two AFP surveillance performance indicators: detection of two or more nonpolio AFP (NPAFP)^†^ cases per 100,000 children aged ≤15 years per year at subnational administrative levels and adequate stool specimen^§^ collection for ≥80% of AFP cases. ES in Afghanistan consists of poliovirus testing of systematically collected sewage samples within all eight regions: in 2023 at 38 sites in 21 districts located in 16 provinces and in 2024, expanded to 43 sites located in 24 districts in 18 provinces.

WHO and UNICEF annually estimate national routine immunization coverage using recent surveys and other data (*7*). SIA effectiveness measures include administrative coverage from data collected during the campaign (doses administered during the campaign divided by the estimated target population) and point estimates of postcampaign monitoring surveys. Administrative data are less reliable but more widely available than are survey data. Postcampaign surveys are conducted by independent, trained personnel who select households by purposive sampling, and monitor finger-marking of children (performed by SIA teams upon OPV administration) as evidence of recent SIA vaccination; the proportions of children who are finger-marked among children in the surveyed households in the target age group are reviewed and analyzed.

### Data Analysis

Vaccination activities, coverage data, and performance indicators were reviewed and tabulated by region and by vaccination modality. AFP and ES data, characteristics of patients with laboratory-confirmed poliomyelitis, and genomic sequence analysis of poliovirus isolates were reviewed and described. Vaccine dose histories reported by caregivers and collected during AFP investigations were reviewed and analyzed among children with confirmed WPV1 cases.

Genomic sequencing analysis of the region coding the VP1 capsid protein of all poliovirus isolates provides critical detail on transmission of genetic lineages and surveillance quality. Genomic analysis was carried out by a WHO regional reference laboratory for poliomyelitis. This activity was reviewed by CDC, deemed not research, and was conducted consistent with applicable federal law and CDC policy.^¶^

## Results

### Immunization Activities

In 2023, WHO/UNICEF estimated national 3-dose coverage with bOPV among children aged 1 year in Afghanistan was 68%, compared with 56% and 61% in 2021 and 2022, respectively; estimated 2-dose IPV coverage was 45%, compared with 30% in 2021 and 43% in 2022. During 2023, the polio program implemented 13 bOPV SIAs targeting children aged <5 years: three national immunization days (NIDs), five subnational immunization days (SNIDs), and five case-response campaigns. Four SIAs focused on the provinces in the East Region and targeted an expanded age group (children aged <10 years). Reported NID administrative coverage ranged from 77% to 98%; SNID coverage ranged from 88% to 97%, and case-response campaigns coverage ranged from 81% to 120% (coverage >100% can occur if the target population is highly underestimated or if vaccine doses are administered to children not in the target age group or area).

During January–July 2024, the polio program implemented two NIDs and three SNIDs; four SIAs were synchronized with neighboring Pakistan. Administrative bOPV coverage during the January–May 2024 SIA ranged from 88% to 97%. In June 2024, authorities announced nationwide house-to-house access for the first time in approximately 7 years. Before nationwide access, house-to-house vaccination was carried out in 72%‒85% of districts, with an increase to 96%‒99% of districts during June and July, which is close to full house-to-house access. After progressively improved campaign access and quality were achieved, in September 2024 authorities abruptly reversed from allowing nationwide house-to-house access to mandating only fixed-post modality.

In 2023, postcampaign monitoring survey coverage ranged from 73% to 85% in the South Region, 80% to 87% in the Northeast, and 90% to 100% in all other regions. Large differences in coverage were noted by delivery modality: coverage among children ranged from 68% to 75% at fixed posts, 68% to 81% at mosques, and 90% to 97% among those reached through house-to-house vaccination. In 2024, SIA coverage determined by postcampaign monitoring surveys increased to 91%–99% in all regions compared with coverage in 2023, except in the South Region, where coverage ranged from 19% to 77% before June when only fixed-site vaccination was approved; during June and July, when house-to-house vaccination was permitted, SIA coverage in the South Region ranged from 89% to 91%. As in 2023, large coverage differences by vaccine administration modality were again noted in 2024, with 73%‒85% fixed-post coverage compared with 90%‒98% house-to-house coverage. OPV was also administered to persons of all ages entering through two main border crossings with Pakistan: Torkham (East Region) and Friendship Gate (South Region). During January 2023‒September 2024, approximately 1.7 million persons were vaccinated. To further improve SIAs and surveillance, both Afghanistan and Pakistan conducted cross-border meetings to enhance coordination, discuss findings, and plan synchronized campaigns.

### Poliovirus Surveillance

**AFP Surveillance.** In 2023, the AFP surveillance system included a network of 1,932 active surveillance sites,** 3,251 passive reporting sites,^††^ and 49,870 community-based reporting volunteers; this network expanded in 2024 to 2,078 active sites, 3,315 passive sites, and 50,409 reporting volunteers. The NPAFP rate in 2023 was 26 per 100,000 children aged <15 years (regional range = 17.0–39.1), and stool specimen adequacy was 94% (regional range = 90.7%–97.0%) (Table). During January–September 2024, the annualized NPAFP rate was 23.7 per 100,000 persons aged <15 years (regional range = 15.2–36.8), and stool adequacy was 95% (regional range = 91.8%–98.0%).

**TABLE:** Acute flaccid paralysis surveillance performance indicators, reported cases of wild poliovirus type 1, and number of environmental specimens with detection of wild poliovirus type 1, by region and period — Afghanistan, January 2023–September 2024*

Region	AFP surveillance performance indicators						No. of WPV1 cases reported				No. of ES samples with WPV1 detected^†^			
	No. of AFP cases		NPAFP rate^§^		% With adequate stool specimens^¶^		2023		2024		2023		2024	
	2023	2024	2023	2024**	2023	2024	Jan–Jun	Jul–Dec	Jan–Jun	Jul–Sep	Jan–Jun	Jul–Dec	Jan–Jun	Jul–Sep
**All**	**5,856**	**4,080**	**26.0**	**23.7**	**94.0**	**94.6**	**5**	**1**	**11**	**12**	**32**	**30**	**55**	**30**
Badakhshan	113	98	17.0	19.3	96.5	98.0	0	0	0	0	0	0	0	0
Central	1,144	597	22.3	15.2	97.0	97.8	0	0	0	0	0	2	1	4
East	885	638	39.1	36.8	93.6	94.4	5	1	2	0	30	17	14	3
North	482	341	17.3	16.3	90.7	92.4	0	0	0	0	1	0	0	0
Northeast	569	413	22.5	21.3	95.4	95.7	0	0	0	0	0	0	0	0
South	1,298	999	33.3	33.5	91.1	91.8	0	0	9	12	1	11	36	23
Southeast	566	366	25.2	21.3	94.3	95.9	0	0	0	0	0	0	2	0
West	799	628	26.5	26.8	95.0	95.7	0	0	0	0	0	0	2	0

**Abbreviations:** AFP = acute flaccid paralysis; ES = environmental surveillance; NPAFP = nonpolio acute flaccid paralysis; WPV1 = wild poliovirus type 1.

* Data as of October 14, 2024.

^†^ Total number of ES samples by period, January 2023–September 2024.

^§^ Cases per 100,000 persons aged <15 years. The surveillance performance indicator target for countries with poliovirus circulation is two or more NPAFP cases per 100,000 persons aged <15 years per year.

^¶^ Percentage of cases with adequate stool specimen. An adequate stool specimen refers to the quantity and quality required to detect poliovirus and is defined as two stool specimens of sufficient quality for laboratory analysis, collected ≥24 hours apart and ≤14 days of paralysis onset, and arriving at a WHO-accredited laboratory in good condition (<8 grams, reverse cold chain maintained, without leakage or desiccation, and with proper documentation).

** Annualized based on AFP surveillance data through September 2024.

**Environmental Surveillance.** In 2023, WPV1 was detected in 62 ES samples from eight provinces in four regions: East (Nangarhar [42] and Kunar [five]), South (Kandahar [eight], Zabul [two], Helmand [one], and Uruzgan [one]), Central (Kabul [two]), and North (Balkh [one]). During January–September 2024, 85 WPV1 ES detections were reported in 10 provinces in five regions: South (Kandahar [35], Helmand [22], and Uruzgan [two]), East (Nangarhar [13], Laghman [three], and Kunar [one]), Southeast (Paktya [one] and Ghazni [one]), Central (Kabul [five]), and West (Hirat [two]).

### Epidemiology of Poliovirus Cases

**FIGURE 1:**
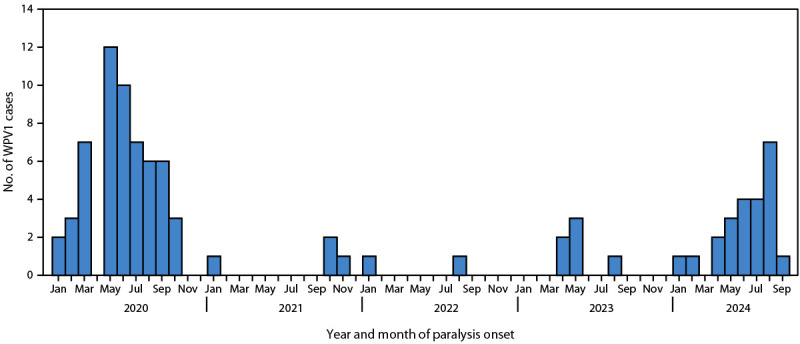
Number of wild poliovirus type 1 cases, by month of paralysis onset (N = 91) — Afghanistan, January 2020–September 2024* **Abbreviation: **WPV1 = wild poliovirus type 1. * As of September 30, 2024.

**FIGURE 2:**
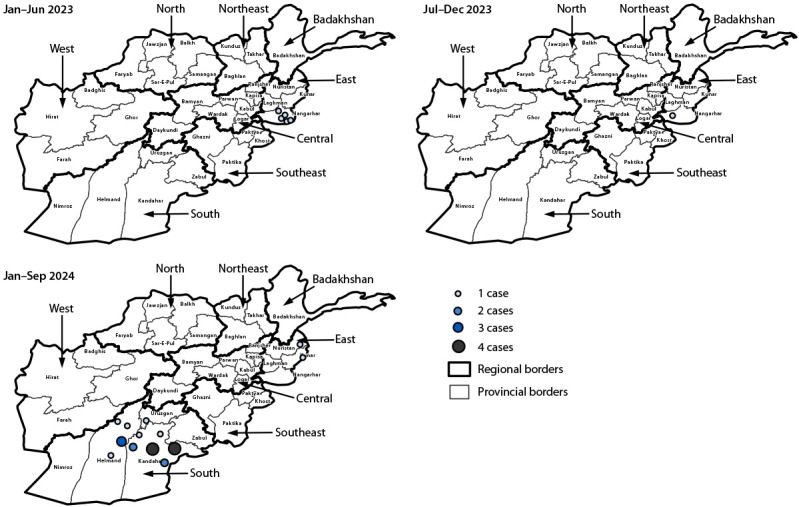
Reported cases of polio caused by wild poliovirus type 1 (N = 29), by region, province, and period — Afghanistan, January 2023–September 2024

During 2021–2022, six WPV1 cases were reported, from the Southeast and Northeast regions. Six total WPV1 cases were reported during 2023, all from Nangarhar province in the East Region (five reported cases during January–June 2023 and one during July–December) (Figure 1) (Figure 2). In 2023, the mean age at onset of paralysis was 79 months (6 years, 7 months) (range = 30–144 months). Caregiver histories indicated that these children had received >7 OPV doses through SIAs and a median of 3 doses through routine immunization (range = 1–3 doses).

During January–September 2024 (as of November 1), 23 WPV1 cases were reported in Afghanistan, representing approximately four times the number of cases during all of 2023, and the highest number in four years (Figure 1). Cases in 2024 were reported from five provinces in two regions: East (Nuristan [one] and Kunar [one]) and South (Kandahar [14], Helmand [six], and Uruzgan [one]). The mean patient age was 39 months (3 years, 3 months) (range = 8‒120 months). Among the 23 WPV1 patients, three had never received an OPV dose through routine immunization or SIAs. Seventeen children received OPV through SIAs (average = 8 doses; range = 2–20), and 17 received OPV through routine immunization (average = 3 doses; range = 1–5).

### Genomic Sequence Analysis of Poliovirus Isolates

Genomic sequence analysis revealed two WPV1 genetic clusters (groups of isolates sharing ≥95% identity) during July 2023–September 2024. A majority of WPV1 isolates, including those from all AFP cases, were part of cluster YB3A4A, found exclusively in the East Region in 2023. Detection of YB3A4A lineages along the southern corridor, including Kandahar and Helmand provinces, occurred in 2024. All isolates from AFP cases were genetically linked to other sequences within Afghanistan, apart from one isolate from an August 2024 case in Kandahar province linked to a Pakistan isolate. Isolates from three ES detections were part of cluster YB3A4B, with a single detection in a sample collected in March 2024 from Hirat province (West Region) and were linked to both Afghanistan and Pakistan isolates. The first ES isolate in the South Region in 2024 was linked to transmission in the East Region in 2023.

Among the 23 AFP WPV1 case isolates identified during January–September 2024, one isolate (from a case detected in Kandahar) was >1.5% divergent from its closest genetic match, the orphan virus threshold,^§§^ and four others, including two from Kandahar, one from Nuristan, and one from Helmand, were >1.1% (but ≤1.5%) divergent. Five ES isolates exceeded the orphan virus threshold, including one from Nangarhar in 2023 and four in 2024, from Kandahar (two), Kabul (one), and Paktya (one). Fourteen other ES isolates, from Helmand (one), Kabul (two), Kandahar (eight), Laghman (one), and Nangarhar (two) were >1.1% divergent from their closest genetic match. The above six orphan viruses (1 AFP, 5 ES) and 18 isolates >1.1% divergent (4 AFP, 14 ES) from their closest genetic match identified during this reporting period indicate a considerable proportion of undetected cases and therefore substantial gaps in surveillance.

## Discussion

During a 2-year period, 2021–2022, Afghanistan reported six WPV1 cases, followed by six cases in 2023. In response to five WPV1 cases reported during the first half of 2023 from Afghanistan’s Nangarhar province, an intense SIA calendar was implemented in the East Region. One of the six 2023 cases was reported in Nangarhar later in 2023. During October 2023–early 2024, approximately 1 million Afghans were forced to return from Pakistan, 44% of whom settled in the South Region, mostly in Kandahar province, and 26% in the East Region, mostly in Nangarhar province. These two provinces, which reported the majority of polio cases during 2023‒2024, had been historical WPV1 reservoirs in Afghanistan (*8*). Despite achieving AFP surveillance performance indicators thresholds, genomic sequencing analyses depicting orphan viruses and others with marked divergence from closest known genetic relatives highlight limitations in the sensitivity of surveillance and likely indicate undetected cases in undervaccinated children. Compared with polio cases in 2023 (*9*), patients in 2024 were younger at onset of paralysis and had received fewer vaccine doses; three 2024 WPV cases were in children who had never received an OPV dose through routine immunization or SIAs.

In Afghanistan, women are allowed to enter homes to vaccinate young children, whereas men who are not close relatives are not. Despite incrementally increased numbers of female workers in the polio program in Afghanistan during the period up to 2021, progressively tighter restrictions on women’s rights and freedom of movement have limited their engagement in polio eradication activities, both as polio workers and as caregivers seeking vaccines for their children outside the home.

In early 2024, the Afghanistan polio program instituted several initiatives that provided desired commodities for hard-to-reach children and strengthened humanitarian organizations’ community engagement in polio eradication activities. After authorities announced nationwide house-to-house access, 99% of children were vaccinated through this modality during June and July SIAs, and vaccination coverage improved in all areas; however, authorities unexpectedly reversed their decision in August. The polio program postponed the September SIA to strategize on how to optimize fixed-site campaign quality. Administrative data and postcampaign–monitoring survey data consistently indicate that SIA coverage achieved through non–house-to-house modalities is unacceptably low. This finding is especially relevant for the South Region, the current epicenter of transmission, where reaching children has always been challenging.

### Limitations

The findings of this report are subject to at least two limitations. First, population figures and campaign target estimates have been based on outdated detailed community vaccination plans, some from 2017. The large influx of returnees from Pakistan during 2023‒2024 exacerbated uncertainties in population estimates. Second, caregiver histories of doses administered might be inaccurate because of poor recall among caregivers and reporting bias among investigators.

### Implications for Public Health Practice

Data indicate that house-to-house vaccination is the most effective campaign modality. The current approach by authorities in Afghanistan to limit SIA implementation to modalities other than house-to-house jeopardizes eradication. Interrupting transmission in the South Region reservoir will require further strengthening of coordination between the Afghanistan and Pakistan polio programs. To ensure that every vulnerable child is reached with vaccine, nationwide house-to-house activities are necessary, as is strengthening the routine childhood immunization program to provide a pathway to global polio eradication.

## Footnotes

* SIAs are immunization activities that supplement the routine immunization program and generally target children aged <5 years for OPV, regardless of their vaccination history. Countries with ongoing polio circulation conduct multiple SIA activities.

† NPAFP cases are discarded as polio cases because they do not have laboratory or other documentation of poliovirus as the cause of paralysis. The expected background NPAFP rate is two or more cases per 100,000 children aged <15 years per year, the WHO standard performance indicator target for sufficiently sensitive surveillance to detect a case of polio.

§ An adequate stool specimen refers to the quantity and quality required to detect poliovirus and is defined as two stool specimens of sufficient quality for laboratory analysis, collected ≥24 hours apart and ≤14 days of paralysis onset, and arriving at a WHO-accredited laboratory in good condition (<8 grams, reverse cold chain maintained, without leakage or desiccation, and with proper documentation).

¶ 45 C.F.R. part 46.102(l)(2), 21 C.F.R. part 56; 42 U.S.C. Sect. 241(d); 5 U.S.C. Sect. 552a; 44 U.S.C. Sect. 3501 et seq.

** Active AFP surveillance involves proactively seeking (weekly or biweekly), reporting, and investigating cases. Active systems tend to be more accurate and comprehensive, although they are more time-consuming than are passive systems.

†† Passive AFP surveillance relies on reporting by providers or other persons; passive AFP is less time-consuming and expensive but tends to be less accurate because of inherent underreporting. Reporting typically occurs monthly.

§§ Orphan viruses are >1.5% divergent from their closest genetic match (i.e., <98.5%) identity and can indicate a gap in poliovirus surveillance. The Global Polio Laboratory Network bases its findings on analysis of the genetic divergences of the 906-nucleotide VP1 capsid protein coding region of poliovirus isolates.
